# Magnetic Resonance Imaging Findings of Lymphoepithelial Carcinoma of the Submandibular Gland: A Case Report

**DOI:** 10.7759/cureus.49939

**Published:** 2023-12-04

**Authors:** Hiroyuki Fujii, Akifumi Fujita, Hiroshi Nishino, Mitsuru Matsuki, Harushi Mori

**Affiliations:** 1 Radiology, Jichi Medical University, School of Medicine, Shimotsuke, JPN; 2 Radiology, Haga Red Cross Hospital, Moka, JPN; 3 Otolaryngology-Head and Neck Surgery, Jichi Medical University, School of Medicine, Shimotsuke, JPN; 4 Radiology, Jichi Children's Medical Center Tochigi, Shimotsuke, JPN

**Keywords:** epstein-barr virus, mri, submandibular gland, salivary gland, lymphoepithelial carcinoma

## Abstract

Lymphoepithelial carcinoma (LEC) is an uncommon neoplasm strongly associated with Epstein-Barr virus (EBV). LEC of the salivary glands (LECSG) is very rare: the most commonly affected site is the parotid gland, followed by the submandibular gland. This report describes the case of a 58-year-old man who presented with a left submandibular mass that had gradually increased in size over five years. On magnetic resonance imaging (MRI), the mass showed low apparent diffusion coefficient (ADC) values, rapid initial enhancement before reaching a plateau on dynamic contrast-enhanced MRI (DCE-MRI), and internal septal-like enhancement. Histopathologically, the tumor comprised polygonal or round tumor cells with atypical or pleomorphic nuclei and numerous lymphocytes, separated by heavy fibrosis. Immunohistological findings were positive for AE/AE3, CD20, and EBV-encoded small RNA in situ hybridization (EBER-ISH), consistent with LEC. A low ADC value with rapid initial enhancement before reaching a plateau on DCE-MRI was thought to reflect abundant cellular components with tumor neoangiogenesis, whereas internal septal-like enhancement reflects separating heavy fibrosis. To the best of our knowledge, this is the first case report describing ADC value and DCE-MRI findings of LECSG, and these findings can be considered characteristic MRI findings of LECSG.

## Introduction

Lymphoepithelial carcinoma (LEC) is an uncommon neoplasm characterized by undifferentiated malignant epithelial cells with extensive non-neoplastic lymphoid infiltration in the stroma [1]. LEC commonly occurs in the nasopharynx, but LEC can arise in the salivary glands, tonsils, thymus, larynx, and soft palate in the head and neck region [2,3]. LEC of the salivary gland (LECSG) is very rare and accounts for 0.3-5.9% of all malignant tumors of the salivary glands [1,4,5]. The majority of LECSGs occur in the parotid gland and rarely in the submandibular gland [6]. To date, there have been few reports describing the imaging findings of LECSG, and, in particular, only one study evaluated the signal intensity of LECSG on conventional magnetic resonance imaging (MRI) sequences [7-10]. Herein, we report an additional case of LEC of the left submandibular gland and discuss its MRI findings. To the best of our knowledge, this is the first case report describing apparent diffusion coefficient (ADC) value and dynamic contrast-enhanced MRI (DCE-MRI) findings of LECSG. 

## Case presentation

A 58-year-old man was referred to the hospital for further examination of a left submandibular mass. He was aware of the left mandibular mass approximately five years earlier, and the mass had gradually increased in size since then. The patient had a history of hypertension, but he was not receiving any medical treatment. At the hospital, a physical examination revealed a mass of 33 mm in size in his left submandibular region, and its mobility was good. No cervical lymph nodes were palpable. On neurological examination, there was no facial paralysis. Laboratory findings were unremarkable.

**Figure 1:**
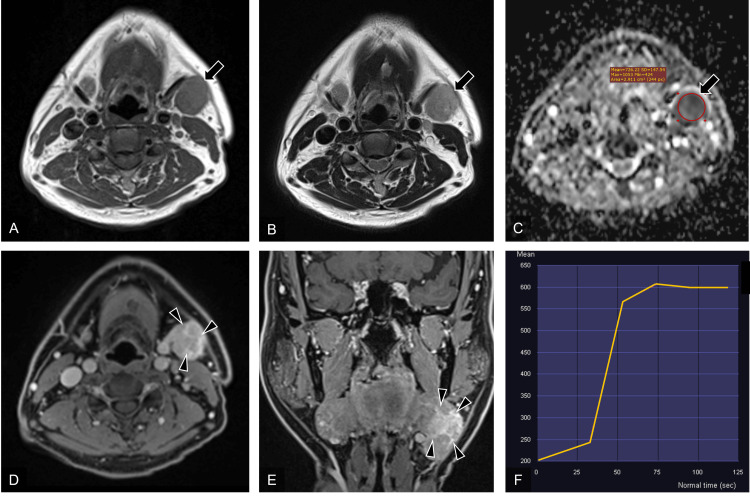
Magnetic resonance images of the mass of the left submandibular gland. T1WI: T1-weighted image; T2WI: T2-weighted image; ADC: apparent diffusion coefficient; Gd-FS-T1WI: gadolinium-enhanced fat-suppressed T1WI; DCE-MRI: dynamic contrast-enhanced magnetic resonance imaging (A) axial T1WI, (B) axial T2WI, (C) ADC map derived from diffusion-weighted images (b = 1000 s/mm^2^), (D) axial Gd-FS-T1WI, (E) coronal Gd-FS-T1WI, (F) time-intensity curve of DCE-MRI. The mass shows moderate signal intensity on T1WI (A, arrow), a slightly high signal intensity on T2WI (B, arrow), and low signal intensity on the ADC map (C, arrow). The ADC value was 0.726 × 10^-3^ mm^2^/s (C). The time-intensity curve shows rapid initial enhancement before reaching a plateau (F). The mass shows internal septal-like enhancement (D, E, arrowheads).

Ultrasonography showed a well-defined, hypoechoic mass measuring 32 × 23 × 29 mm^3^ bordering the submandibular gland. Color Doppler showed abundant blood flow within the mass. No obvious calcification was observed. On MRI, the mass showed moderate signal intensity on T1-weighted images (T1WI) and slightly high signal intensity on T2-weighted images (T2WI) (Figures 1A, 1B). The ADC value was 0.726 × 10^-3^ mm^2^/s (Figure 1C). On DCE-MRI, the mass showed rapid initial enhancement before reaching a plateau (Figure 1F). Gadolinium-enhanced fat-suppressed T1WI (Gd-FS-T1WI) showed stronger enhancement than the adjacent normal submandibular gland tissue, with internal septal-like enhancement (Figures 1D, 1E).

**Figure 2:**
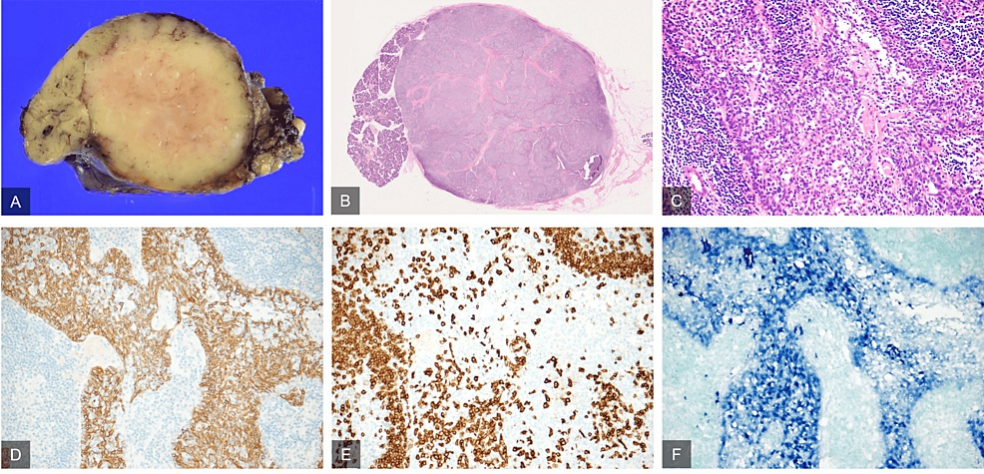
Histopathological and immunohistological findings EBER-ISH: Epstein-Barr virus-encoded small RNA in situ hybridization (A) formalin-fixed specimen, (B, C) hematoxylin-eosin staining, (D) cytokeratin AE1/AE3 staining, (E) CD20 antibody staining, (F) EBER-ISH. Gross pathology shows a well-defined, non-encapsulated, tan-white mass (A). Microscopically, there are large nests and lobules of tumor separated by heavy fibrosis (B). The tumor is comprised of polygonal or round tumor cells with atypical or pleomorphic nuclei and numerous lymphocytes (C). The tumor cells are positive for AE/AE3, CD20, and EBER-ISH (D-F), consistent with lymphoepithelial carcinoma.

No cervical lymphadenopathy was noted. A fine needle biopsy was performed, and its Milan system category was class 3, with abundant lymphocytes and a few epithelial-like cell clusters and stromal cells seen on a hematogenous background. The patient underwent a left submandibular adenectomy for diagnostic and therapeutic purposes. Intraoperative findings showed that the mass was located posteroinferiorly within the submandibular gland and showed mild adhesions to the surrounding tissues. Gross pathology showed a well-defined, non-encapsulated, tan-white mass (Figure 2A). Microscopically, the tumor consisted of polygonal or round tumor cells with atypical or pleomorphic nuclei and numerous lymphocytes with lymph follicles (Figure 2C). Tumor cells formed foci, arranged in sheets, with separating heavy fibrosis (Figure 2B). Immunohistological findings were positive for AE1/AE3, cytokeratin 5, p53, and CD20 (Figures 2D, 2E). The positivity rate for Ki67 was 30-40%. Epstein-Barr virus (EBV)-encoded small RNA in situ hybridization (EBER-ISH) was positive (Figure 2F).

These findings were consistent with LEC. The patient underwent postoperative radiation therapy using intensity-modulated radiation therapy with 60 Gy in 30 fractions. He then developed mild mandibular marginal branch paralysis immediately after surgery, but later recovered. There was no recurrence at the evaluation one year and six months after surgery.

## Discussion

LEC was first described by Hilderman et al. in 1962 as the malignant transformation of benign lymphoepithelial lesions [11]. The vast majority of LECs have occurred in Southeast Asian and Arctic Inuit populations, as well as descendants of these ethnic groups who migrate to nonendemic countries [12,13]. Previous studies have described the strong association of EBV with LECSG in endemic areas [13]. The present case was also positive on EBER-ISH. Although not fully elucidated, several studies have demonstrated that EBER induces insulin-like growth factor, which acts as an autocrine growth factor, suggesting that EBV infection directly affects the pathogenesis of EBV-associated malignant lesions, such as gastric carcinoma and nasopharyngeal carcinoma [6].

In a literature review of LECSG, no sex predilection was identified [13]. Patients with LECSG have a wide age range, from 10 to 86 years, with an average age of 47.1 years and a median age of 46.0 years. Nonendemic patients tend to be older than endemic patients [13,14]. LECSG occurs most frequently in the parotid glands (76.4%), followed by the submandibular glands (17.2%), sublingual glands (0.5%), and minor salivary glands (5.9%). Clinical presentations of LECSG are usually nonspecific; patients with LECSG present with parotid swelling or growth of a mass. Tumor size ranges from 0.7 to 15 cm, with an average size of 3.9 cm and a median size of 3.8 cm. Cervical lymphadenopathy was found in 17% of the patients.

Fine needle aspiration cytology (FNAC) is a rapid, nonsurgical diagnostic technique that can establish the definitive diagnosis of a salivary gland lesion. The diagnostic accuracy of FNAC in the diagnosis of salivary gland lesions ranges from 85% to 96% [15]. However, its diagnostic accuracy for LECSG was 78.6% [16]. In fact, FNAC could not lead to a correct diagnosis in the present case. Therefore, we believe that computed tomography (CT) and MRI are still valuable in the preoperative evaluation and biopsy guidance for LECSG.

Wang et al. reported CT and MRI findings of 56 patients with a solitary LECSG [7]. For the tumor margin, a well-defined margin was the most common (42.9%), followed by partially defined (39.3%) and ill-defined (17.9%). For the morphological pattern, lobular shape was the most common (60.7%), followed by round or oval (30.4%) and irregular (8.9%). Calcification was seen in 10.7%. As for the interior of the mass on unenhanced CT, 71.4% showed homogeneous density, and the remaining 28.6% showed heterogeneous density. Contrast enhancement was seen in 87.5%. The authors concluded that a solitary LECSG usually appears as an enhanced and homogeneous mass with a lobulated shape and well-defined or partially defined margins. Of note, MRI signal intensity was not investigated in this study.

To date, there has been only one report describing MRI signal intensity of six LECSG cases (parotid glands in four patients, submandibular gland in one, and sublingual gland in one) [9]. On T1WI, one patient had slightly high signal intensity, and five patients had iso-signal intensity. On T2WI, four patients had low signal intensity, and three patients had slightly high signal intensity. After the administration of a contrast agent, one patient showed poor contrast enhancement, four patients showed moderate contrast enhancement, and one patient showed intense enhancement. In the present case, LECSG showed moderate signal intensity on T1WI and slightly high signal intensity on T2WI, consistent with the previous report [9]. On Gd-FS-T1WI, internal septal-like gadolinium enhancement was seen, reflecting separating fibrosis on histopathological examination.

To the best of our knowledge, there have been no reports describing the ADC value and DCE-MRI findings of LECSG. In the present case, the ADC value was low (0.726 × 10^-3^ mm^2^/s), and DCE-MRI showed rapid initial enhancement before reaching a plateau. A low ADC value with rapid initial enhancement before reaching a plateau on DCE-MRI is thought to reflect an abundant cellular component with tumor neoangiogenesis [17,18]. In the present case, the internal septal-like gadolinium enhancement was considered important in differentiating LECSG from other malignant salivary gland tumors.

## Conclusions

A case of LEC of the left submandibular gland was presented, and its MRI findings were discussed. The LECSG showed a low ADC value, and DCE-MRI showed rapid initial enhancement before reaching a plateau and internal septal-like gadolinium enhancement. To the best of our knowledge, this is the first case report describing ADC value and DCE-MRI findings of LECSG, and these findings can be considered characteristic MRI findings of LECSG. 
